# “Enheduanna—A Manifesto of Falling” Live Brain-Computer Cinema Performance: Performer and Audience Participation, Cognition and Emotional Engagement Using Multi-Brain BCI Interaction

**DOI:** 10.3389/fnins.2018.00191

**Published:** 2018-04-03

**Authors:** Polina Zioga, Frank Pollick, Minhua Ma, Paul Chapman, Kristian Stefanov

**Affiliations:** ^1^Interactive Filmmaking Lab, School of Computing and Digital Technologies, Staffordshire University, Stoke-on-Trent, United Kingdom; ^2^Perception Action Cognition Lab, School of Psychology, University of Glasgow, Glasgow, United Kingdom; ^3^School of Simulation and Visualisation, Glasgow School of Art, Glasgow, United Kingdom

**Keywords:** live brain-computer cinema performance, brain-computer interface (BCI), multi-brain interaction, electroencephalography (EEG), performer, audience participation, attention, emotional engagement

## Abstract

The fields of neural prosthetic technologies and Brain-Computer Interfaces (BCIs) have witnessed in the past 15 years an unprecedented development, bringing together theories and methods from different scientific fields, digital media, and the arts. More in particular, artists have been amongst the pioneers of the design of relevant applications since their emergence in the 1960s, pushing the boundaries of applications in real-life contexts. With the new research, advancements, and since 2007, the new low-cost commercial-grade wireless devices, there is a new increasing number of computer games, interactive installations, and performances that involve the use of these interfaces, combining scientific, and creative methodologies. The vast majority of these works use the brain-activity of a single participant. However, earlier, as well as recent examples, involve the simultaneous interaction of more than one participants or performers with the use of Electroencephalography (EEG)-based multi-brain BCIs. In this frame, we discuss and evaluate “Enheduanna—A Manifesto of Falling,” a live brain-computer cinema performance that enables for the first time the simultaneous real-time multi-brain interaction of more than two participants, including a performer and members of the audience, using a passive EEG-based BCI system in the context of a mixed-media performance. The performance was realised as a neuroscientific study conducted in a real-life setting. The raw EEG data of seven participants, one performer and two different members of the audience for each performance, were simultaneously recorded during three live events. The results reveal that the majority of the participants were able to successfully identify whether their brain-activity was interacting with the live video projections or not. A correlation has been found between their answers to the questionnaires, the elements of the performance that they identified as most special, and the audience's indicators of attention and emotional engagement. Also, the results obtained from the performer's data analysis are consistent with the recall of working memory representations and the increase of cognitive load. Thus, these results prove the efficiency of the interaction design, as well as the importance of the directing strategy, dramaturgy and narrative structure on the audience's perception, cognitive state, and engagement.

## Introduction

The fields of neural prosthetic technologies and Brain-Computer Interfaces (BCIs) have witnessed in the past 15 years an unprecedented development (Wolpaw and Wolpaw, 2012; He et al., 2013), bringing together theories and methods from the areas of signal processing, machine learning (pattern recognition), computational intelligence, neuroscience, statistics, linear algebra but also digital media and the arts. Accordingly, the interdisciplinary applications of BCIs span across health purposes for patients, purposes for healthy populations in their working environment, as well as entertainment and the arts (He et al., 2013; Bamdad et al., 2015). However, the latter are often viewed with scepticism. Historically, ideas such as that art is useful in order to disseminate scientific theories, but not to be used itself as a methodological approach in the pursuit of scientific evidence, or that the creative process cannot be examined scientifically, have been rather disseminated (Belk, 1966). Nevertheless, more recently the cross-fertilisation of different disciplines, working together in the frame of interdisciplinary studies and new movements, is leading the use of scientific theories and methods in the creative process, and creative approaches in the scientific explorations. In the frame of BCIs, artists have been amongst the pioneers of the design of BCI applications since their emergence in the 1960s (Nijholt, 2015), pushing the boundaries of applications in real-life contexts. One year after the first demonstration of an EEG-based BCI, Alvin Lucier presented the *Music For Solo Performer* (1965), which is considered the first performance using the EEG technology. Other pioneering composers, artists, and performers soon followed. With the new research, advancements, and since 2007, the new low-cost commercial-grade wireless devices, there is a new increasing number of interdisciplinary practices, like computer games, interactive installations, and performances that involve the use of these interfaces, combining scientific, and creative methodologies. The vast majority of these works use the brain-activity of a single participant. However, earlier, as well as recent examples, involve the simultaneous interaction of more than one participants or performers with the use of EEG-based multi-brain BCIs (Nijholt, 2015; Zioga et al., 2016). This trend coincides with the increasing interest in the fields of neuroscience and experimental psychology “in studying the mechanisms, dynamics, and processes of the interaction between multiple subjects and their brain-activity” (Zioga et al., 2015) and even more in a real-life context, away from the lab.

In this frame, we discuss and evaluate *Enheduanna—A Manifesto of Falling*, a live brain-computer cinema performance—that is an interactive performative work that combines live, mediatised representations, more specifically live cinema, and the use of BCIs (Zioga et al., 2015, 2016). The performance was realised as a complete combination of scientific and creative practice-based methodologies and solutions, addressing the challenges of the design and implementation of multi-brain BCIs in mixed-media performances with audience participation. It enables for the first time the simultaneous real-time multi-brain interaction of more than two participants, including a performer and members of the audience, using a passive EEG-based BCI system in the context of a mixed-media performance. In passive BCI systems the outputs are derived from the “arbitrary brain activity” taking place “without the purpose of voluntary control”; in contrast to “active” BCIs that can be controlled by the user consciously i.e. with Motor Imagery; and the “reactive” BCIs that derive their outputs from “brain activity arising in reaction to external stimulation,” such as the P300 BCIs (Zander et al., 2008). The project involves a large multidisciplinary team of professionals, performers and public audiences. The premiere took place at the Theatre space of CCA: Centre for Contemporary Arts Glasgow, UK, in July 2015 (Zioga et al., 2016). The performance was also realised as a neuroscientific study in a real-life setting. The first demonstrations and the implementation of the passive EEG-based BCI system within the real-life conditions of a public event enabled the collection of valuable data from seven participants, one performer and two different members of the audience for each performance: qualitative through the completion of questionnaires and quantitative through the recording of their EEG data. The results reveal that the majority of the audience participants, and the performer participant across the majority of the events, were able to successfully identify whether their brain-activity was interacting with the live video projections or not. Additionally, a correlation has been found between their answers to the questionnaires, the elements of the performance that they identified as most special, and the audience's indicators of attention and emotional engagement. Also, the results obtained from the performer's data analysis are consistent with the recall of working memory representations and the increase of cognitive load.

Thus, these results prove the efficiency of the interaction design, as well as the importance of the directing strategy, dramaturgy, and narrative structure on the audience's perception, cognitive state and engagement. The article presents the materials and methods used (section Materials and Methods); followed by the analysis of the participants' behavioural data, and the analysis of the participant's EEG data (section Results). The aforementioned lead and inform a critical discussion of the results (section Discussion), together with additional observations that were made during the live events, and in comparison with important studies and dominant positions on the cognitive experience and engagement of spectators during live performative works and free viewing of films.

## Materials and methods

### Participants

The participants of the study were recruited directly by the first author in the close geographical proximity of the event and included one performer and six members of the audience, two for each performance. The study was approved by the Glasgow School of Art Research Ethics Committee and all participants gave written informed consent. The inclusion criteria were female and male general adult population, aged 18–65 years old, not suffering from a neurological deficit, nor receiving psychiatric or other neurological medication (Zioga et al., 2016). Alongside their demographic data, the participants were asked to complete the “Edinburgh Handedness Inventory” (Oldfield, 1971), in order to determine whether they are right- or left-handed, as this can indicate differences in the underlying mechanisms of the neural processes. More specifically, the performer participant was an ambidextrous, 36 years old female, whose mother tongue is Greek. The six audience participants included (Table 1): three right-handed females, with a mean age of 32.67 years and range 25–43 years; and three right-handed males, with a mean age of 29.67 years and range 28–33 years. Regarding their mother tongues, one audience participant has stated Danish, two English, one Romanian and two Spanish. Whereas no audience participants stated French and Greek as their mother tongues, which were two out of the three languages in which the work was performed, while the third one was English.

**Table 1:** Overall demographic data for the audience participants.

**Audience participants**		**Female**	**Male**	**Total**
Number		3	3	6 (100%)
Age	Mean	32.67	29.67	31.17
	Median	30.00	28.00	29.00
Mother tongue	Danish	1	0	1 (16.67%)
	English	1	1	2 (33.33%)
	French	0	0	0 (0%)
	Greek	0	0	0 (0%)
	Romanian	1	0	1 (16.67%)
	Spanish	0	2	2 (33.33%)
Handedness	Right	3	3	6 (100%)
	Ambidextrous	0	0	0 (0%)
	Left	0	0	0 (0%)

All participants were asked not to consume high caffeine drinks, cigarettes and alcohol and avoid the recreational use of drugs for at least 12 h prior to the study, as these substances can affect the brain's electrical activity (brain waves) and therefore the EEG recordings. The audience participants arrived at the space ~60 min prior to the start of the event, when the venue was closed for the public, in order to answer a preliminary brief questionnaire and start the preparation. When this was completed, the space opened to the general public, they were asked to watch the performance like any other spectator and complete in the end a final brief questionnaire. A similar process was also followed for the actress (Zioga et al., 2016).

### EEG data acquisition

**Figure 1:**
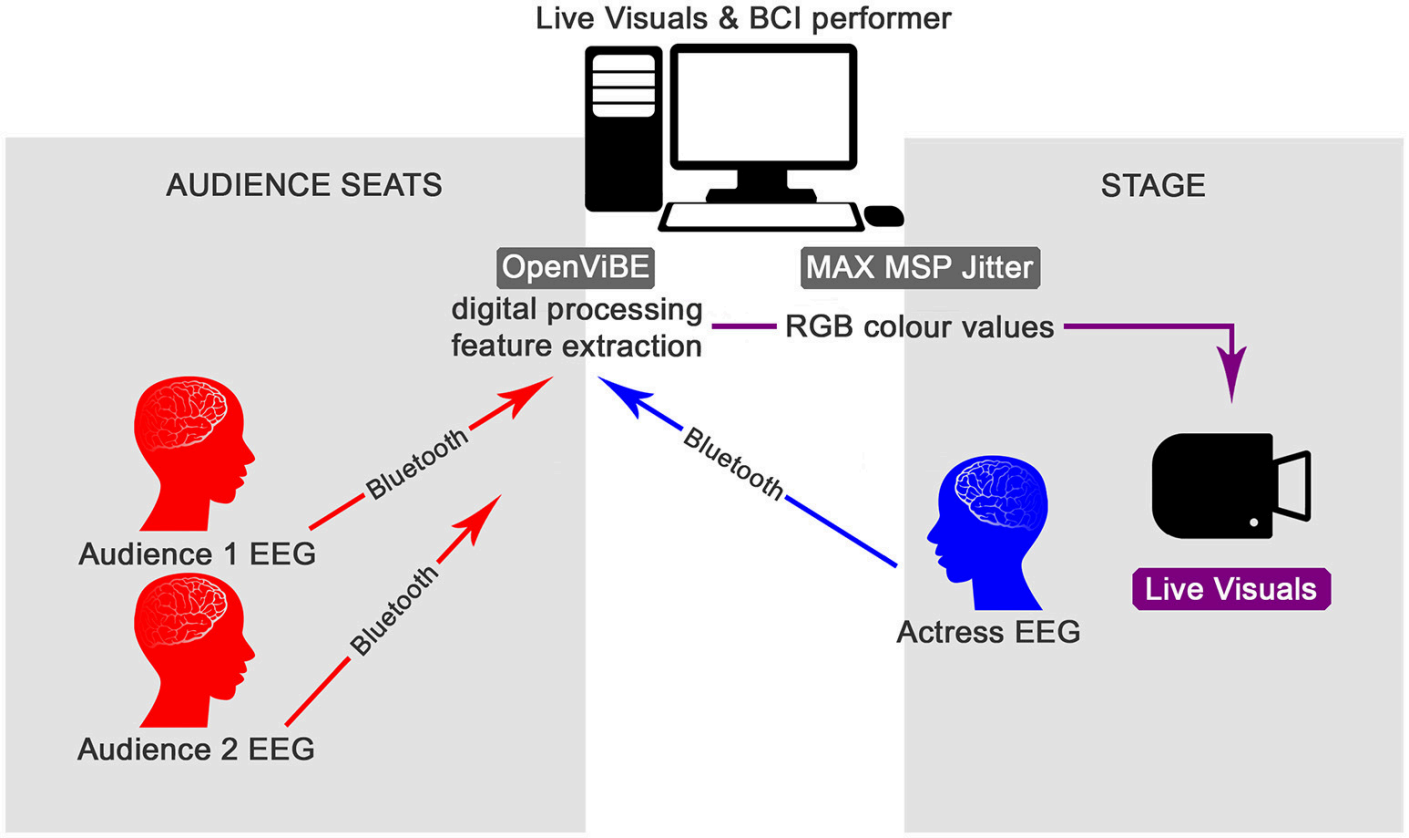
The passive multi-brain EEG-based BCI system. (Images of human heads originally designed by Freepik). Reproduced with permission from Zioga et al. (2016).

The system used during the performance consists of the participants, the performer and the audience members; the data acquisition; the real-time EEG data processing and feature extraction; and the MAX MSP Jitter programming (Figure 1). The participants' raw EEG data are acquired simultaneously with the MyndPlay Brain-BandXL EEG Headset, which has two dry sensors located in the prefrontal cortex, with one passive (Fpz), one active (Fp1) and a grounding electrode on the ear lobe. The data are transmitted wirelessly to the main computer via Bluetooth (Zioga et al., 2015, 2016). The real-time digital recording and processing is performed with the OpenViBE software (Renard et al., 2010) using algorithms that follow the frequency analysis method and a custom-based feature extraction. The methodology focuses on the oscillatory processes of the brain activity, which occur when large neural populations are not engaged with a specific task and synchronise following an oscillatory pattern. While, with the use of temporal band-pass filters, the 4–40 Hz frequencies are processed and sent to the MAX MSP Jitter software. There, the processed EEG values of each participant are scaled to RGB colour values. The blue colour is controlled by the theta frequency band (4–8 Hz); the green by the alpha band (8–13 Hz); and the red colour by the beta (13–25 Hz) and lower gamma frequency (25–40 Hz) bands. The resulting values from the different participants during different parts of the event feed into a “swatch” that provides 2-dimensional RGB colour selection and display, creating a constantly changing single colour, which is then applied as a filter to pre-rendered black and white video files reproduced in real-time. This way, the transition of the participants from relaxed to more alert cognitive states is visualised in the colour scale of the live video-projections as a shift from colder to warmer tints, setting the atmosphere of the visuals but also of the theatrical stage, within the interactive storytelling and the narrative structure (Zioga et al., 2016).

### Experimental conditions and creative methods

The thematic idea of the performance explores the life and work of the historical figure of Enheduanna (ca. 2285–2250 B.C.E.), an Akkadian Princess, the first documented High Priestess of the deity of the Moon Nanna in the city of Ur (present-day Iraq), who is regarded as possibly the first known author and poet in the history of human civilisation, regardless of gender. The concept of the performance is based on the first author's aesthetic, visual, and dramaturgical vision as the director of the work; the thematic idea and the inter-text devised by the actress; and the design and implementation of a new passive multi-brain EEG-based BCI system (Zioga et al., 2016).

More specifically, the performance consists of two parts, introduced to the audience with vignettes projected during the live visuals. During part 1, titled “Me,” we are introduced and immersed in the story of Enheduanna, with the interaction based solely on the actress' brain-activity. This is further divided in scene 1 “Me Transmitting Signals” with an approximate duration of 13 min 14 s, scene 2 “Me Rising” with an approximate duration of 8 min 42 s, and scene 3 “Me Falling” with approximate duration of 10 min 14 s. During part 2, titled “You/We,” the interaction is based first on either of the two audience members' brain-activity (scene 4 “You Measuring the F-Scale” with approximate duration of 5 min 00 s). Whereas, in the second half (scene 5 ‘We’ with approximate duration of 9 min and 43 s), the real-time brain-interaction of the actress and the audience member gradually merges and their averaged values control the colour filter applied to the live video stream. As a result, the narrative and dramaturgical structure brings together the thematic idea of the performance with the use of the interaction technology in a coherent and comprehensive manner and by this it also serves as an evidence of liveness (Zioga et al., 2016).

## Results

### Participants' behavioral data analysis

The collected data from the pre- and post-performance questionnaires aimed to reveal:

1. whether the participants had a prior knowledge or experience using a Brain-Computer Interface device (Table 2) that could possibly influence their participation in the study;
2. whether the participants were able to identify when and how their brain-activity was controlling the live video projections (Table 2); and
3. what were the most special elements of the performance (Table 3).

More specifically, in the frame of the pre-performance preliminary questionnaires the participants were asked: *Do you have a prior knowledge or experience of using a Brain-Computer Interface device? If yes, please give more information i.e. the type/model or device used, the mental tasks performed etc*. The majority of the audience participants (83.33%) replied that they had no prior experience. One audience participant (16.67%) replied that he previously tried out for a few minutes a similar device, as part of a demo presented in a conference, but he didn't know anything about the device. Whereas, the performer participant replied that her only prior experience has been wearing the BCI device during the rehearsals of the currently discussed study (Table 2). Therefore, it is concluded that the participants' prior knowledge did not influence their participation in the study and their responses to the post-performance experience questionnaires.

**Table 2:** Quantitative analysis of participants' answers to pre- and post-performance questions.

	**BCI Knowledge**^a^			**BCI Interaction Awareness**^b^				
	**No**	**Not significant**	**Yes**	**True positive**	**True negative**	**False positive**	**False negative**	**N/A**
**PERFORMER PARTICIPANT**								
1st event	0	1	0	0	0	1	0	0
2nd event	0	1	0	1	0	0	0	0
3rd event	0	1	0	1	0	0	0	0
Total	0 (0%)	3 (100%)	0 (0%)	2 (66.67%)	0 (0%)	1 (33.33%)	0 (0%)	0 (0%)
**AUDIENCE PARTICIPANTS**								
Female	3	0	0	2	0	1	0	0
Male	2	1	0	1	1	0	0	1
Total	5 (83.33%)	1 (16.67%)	0 (0%)	3 (50%)	1 (16.67%)	1 (16.67%)	0 (0%)	1 (16.67%)

a *Do you have a prior knowledge or experience of using a Brain-Computer Interface device? If yes, please give more information i.e. the type/model or device used, the mental tasks performed etc*.

b Did you think/understand that your brain-activity was interacting with the audio and videos during the performance? If yes, when? And how?

**Table 3:** Descriptive analysis of participants' answers to post-performance questions.

	**Performer participant (total events 3)**	**Audience participants (total part. 6)**
**Task** (*What did you do during the study?*)	**Number of events/answer**	**Number of part./answer**
*Watching the performance*		6
*Performing*	3	
*Concentrating in order to read the supertitles*		2
**Mental Strategy** (*Did you use any specific strategy related to mental tasks*?)		
*No specific strategy*	3	4
*Consciously staying engaged*		2
*Focusing especially in part 2 “You”*		1
*Imagining pleasant activities*		1
**Special Impression** (*Was there anything special in the performance for you?*)		
*The whole experience/The whole performance*	2	1
*The relation of the acting experience to the colours of the visuals as an acting environment/The visuals and the changing colours*	1	2
*Part 2 'You/We' of the performance*		2
*The different languages of the texts*		2
*The 'moving' texts*		2
*Feeling 'connected' through the colours of the visuals and the sounds*		1
*The physical control of the performer*		1
**Significant BCI Interaction Awareness Factors** (*When and how did you think/understand that your brain-activity was interacting?*)		
*The changing colours of the visuals*	1	3
*Part 2 'You/We' of the performance*		3
*The explanatory texts in the visuals*		2
*The use of lights*		1
*When not focusing on acting and paying attention to the environment*	1	

Additionally, the participants were asked after the performance: *Did you think/understand that your brain-activity was interacting with the audio and videos during the performance? If yes, when? And how?* As described in section EEG Data Acquisition, in each one of the three events, the brain-activity of both the audience participants was being recorded and processed, but one of them was manually selected in order to generate the RGB colour filter during scene 4 and scene 5. Additionally, the brain-activity of the performer participant was generating the RGB colour filter during scene 1–3 and then jointly with the audience during scene 5. Based on this, the answers of the participants can be categorised as true positive, true negative, false positive, and false negative. As demonstrated more specifically in Table 2, the majority of the audience participants (66.67%) were able to successfully identify whether their brain-activity was interacting with the live visuals (true positive answers) or not (true negative answers). Whereas, one audience participant gave a false positive answer (16.67%) and one was not able to determine (16.67%). The performer participant was 66.67% successful in identifying that her brain-activity was interacting with the live visuals (true positive answers), compared to 33.33% of false positive answers.

In the frame of the post-performance questionnaires the participants were asked what they did during the study, in order to verify that they followed the instructions for participation in the study (Table 3). All the audience participants replied that they watched the performance and the actress replied that she performed, as expected. Additionally, two audience participants reported that they were concentrating on reading the supertitles. The participants were also asked whether they used any specific strategy related to mental tasks, in order to identify any particular factor that might have influenced their cognitive state during the study (Table 3). The majority, the actress in all three events and four out of the six audience participants, did not report any specific strategy. Interestingly, two of the audience participants mentioned that they were consciously staying engaged, one that was focusing especially in part 2 “You” and one that was imagining pleasant activities. None of these though are considered to have any particular influence in their cognitive states during the study.

Another post-performance question that offered valuable insight and feedback was whether there was something in the performance that made a special impression on the participants. Apart from the experience as a whole for both the actress, as well as the audience, the most highlighted elements also include the live visuals and the colours, part 2 “You/We” of the performance, the use of different languages and the “moving” texts. Other elements include the feeling of being “connected” through the colours and the sounds and the physical control of the performer.

Additionally, the participants that were able to successfully identify that their brain-activity was interacting with the live video projections (Table 2) in the questions *If yes, when? And How?*, they highlighted as main factors the changing colours of the visuals, part 2 “You/We” and the explanatory vignettes. Other factors include the use of lights, whereas the actress reported that she was able to understand that her brain-activity was interacting with the video projections when she was not focusing on acting and was able to pay more attention to the environment.

### Participants' EEG data analysis and statistical methods

The participants' raw EEG data were also collected, the quantitative and statistical analysis of which most interestingly revealed a correlation with their answers to the questionnaires. The raw EEG data from all participants were processed offline in the MATLAB R2016b software (MathWorks, 2016) with the EEGLAB 13.6.5b interactive toolbox (Swartz Center of Computational Neuroscience University of California San Diego, 2016) and the IBM SPSS software [IBM (no date)]. During the process a series of challenges and issues have been encountered, mainly due to the particularly long recorded datasets and their resulting increased size. The first challenge was the required increased computational power in order to perform the analysis. In order to address this, the datasets were down-sampled from 512 to 256 Hz rate. Nevertheless, this did not have an effect on the quality of the analysis, since according to Nyquist theorem a sufficient sampling rate is equal to 2 times of the highest frequency of the data (Baraniuk, 2007), which in the case of the currently discussed study is 40 Hz. Following the down-sampling, the datasets were filtered using a windowed sinc Finite Impulse Response (FIR) filter, with cutoff frequency 4–40 Hz, bandpass filter type blackman window type and transition bandwidth 1 (Smith, 1997). Additionally, all the recorded data from all three events included a time-period at the beginning prior to the performance. These initial data were removed and saved, then used in the analysis as the participants' brain-activity baseline, in order to compare it with their brain-activity during the performance.

**Figure 2:**
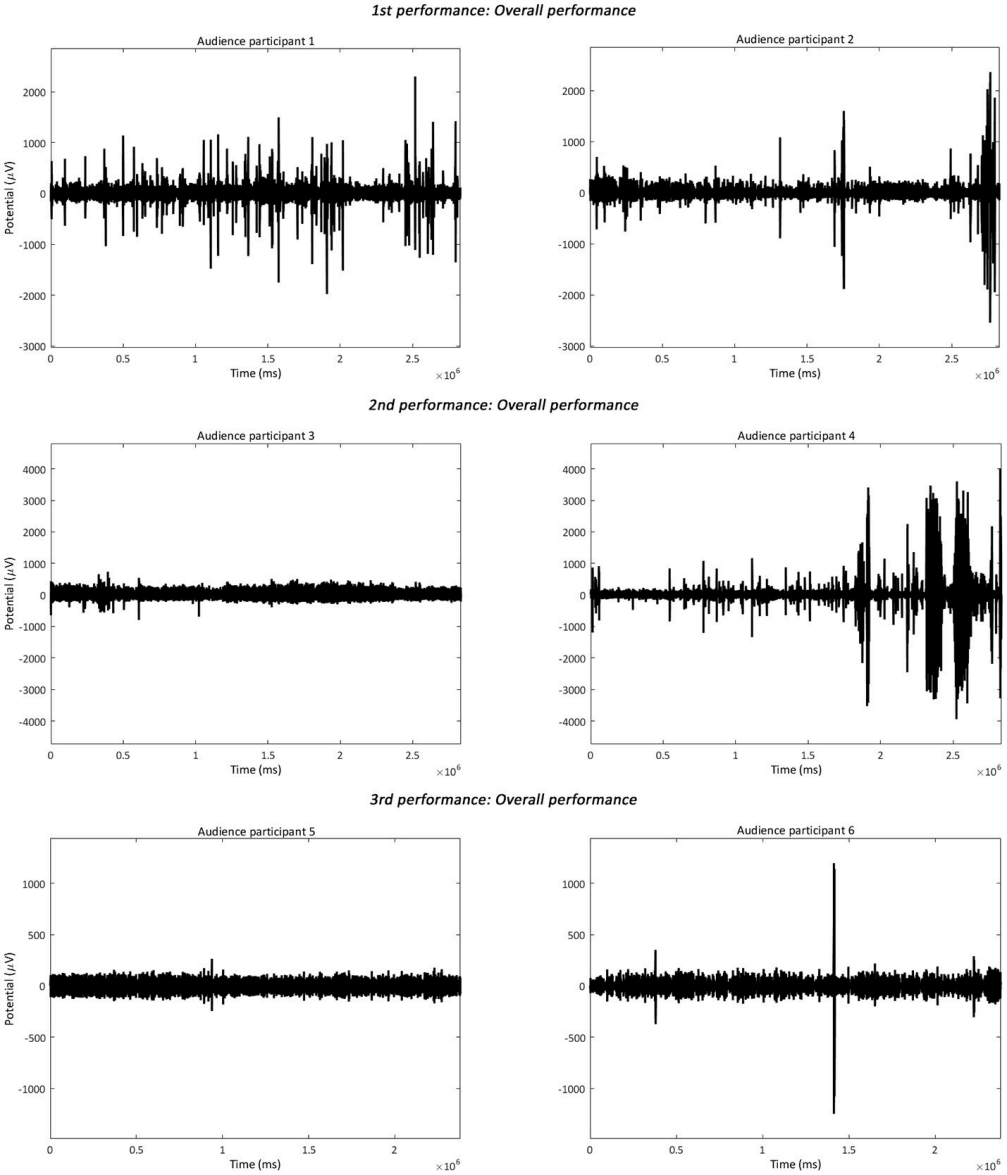
The plotted 4–40 Hz Signal Potential (μV) in the Time Domain (ms) of the audience participants during the overall performance of each performance.

**Figure 3:**
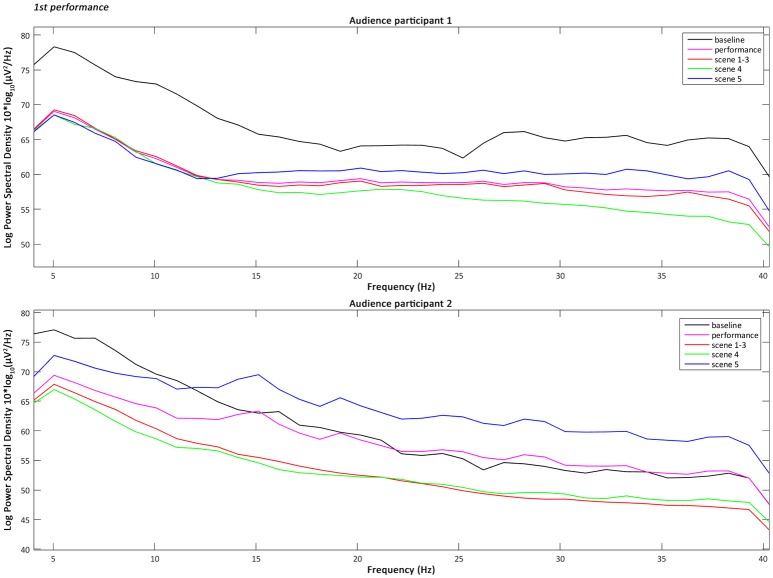
The plotted Power Spectral Density for the 4–40 Hz frequency range of the audience participants 1 and 2 during the baseline, the overall performance, scenes 1–3, scene 4, and scene 5 of the first performance.

**Figure 4:**
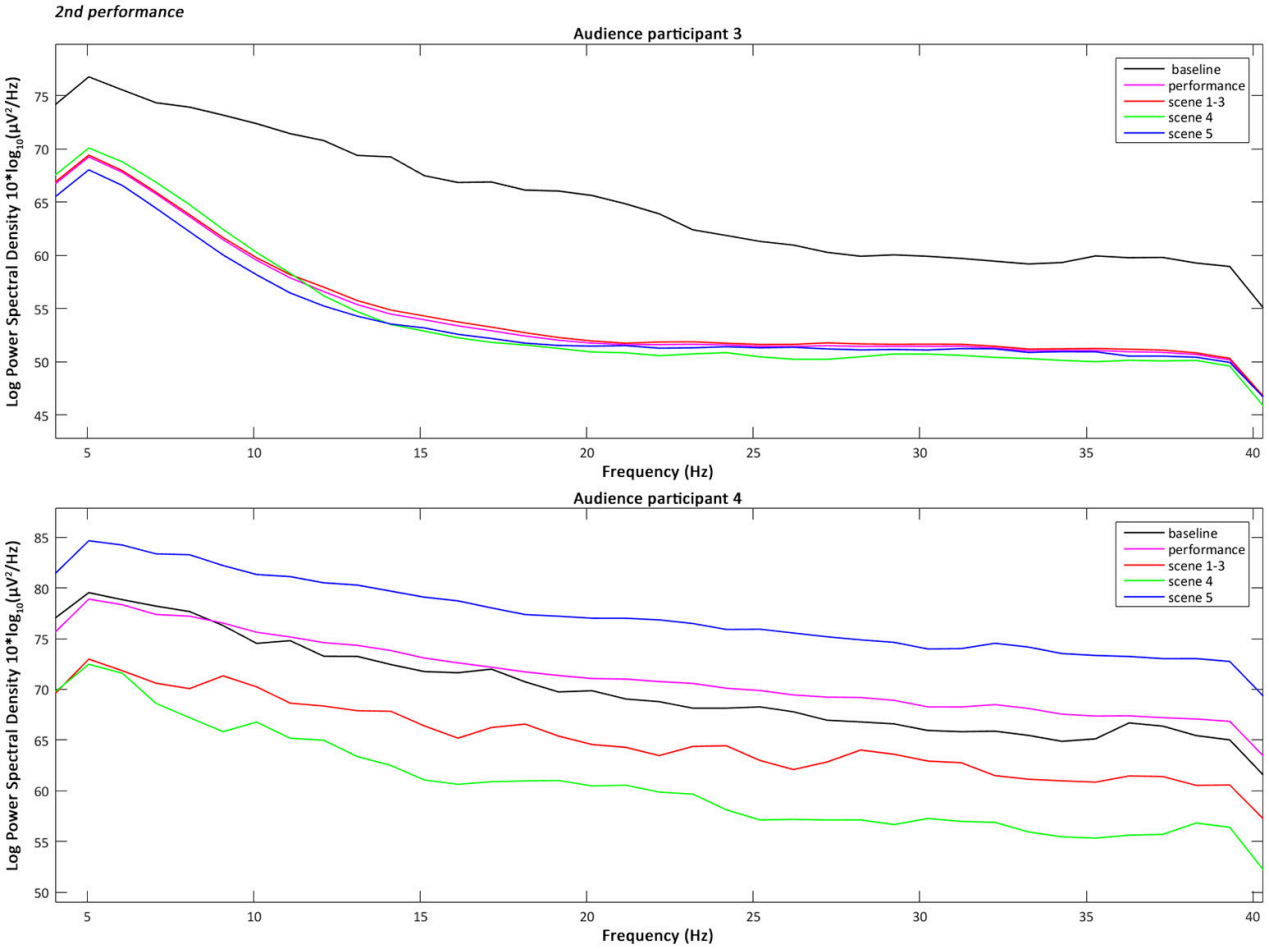
The plotted Power Spectral Density for the 4–40 Hz frequency range of the audience participants 3 and 4 during the baseline, the overall performance, scenes 1–3, scene 4, and scene 5 of the second performance.

**Figure 5:**
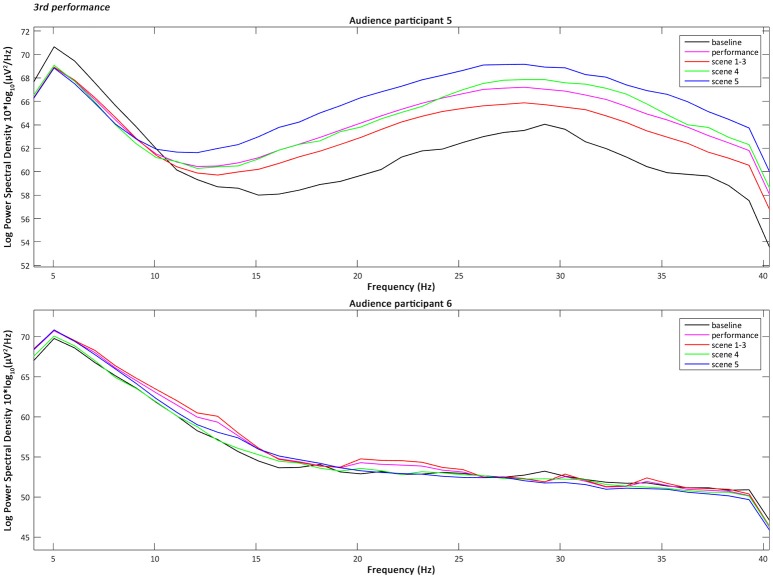
The plotted Power Spectral Density for the 4–40 Hz frequency range of the audience participants 5 and 6 during the baseline, the overall performance, scenes 1–3, scene 4, and scene 5 of the third performance.

**Figure 6:**
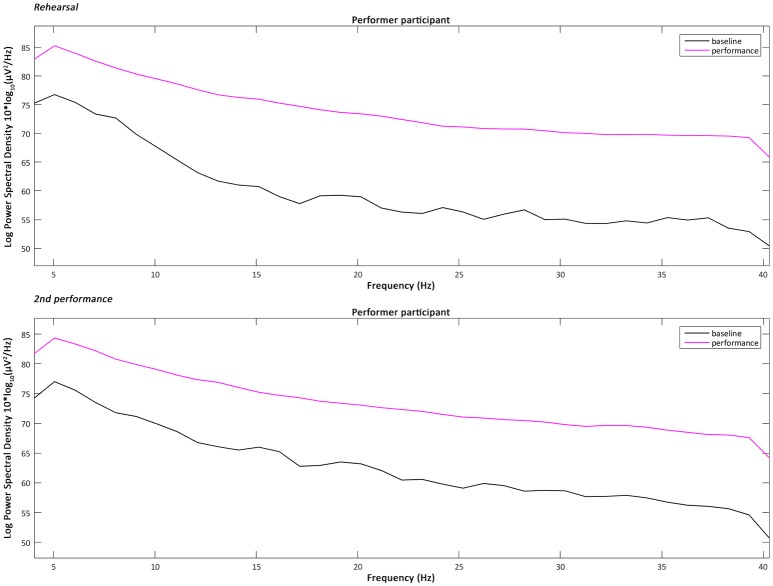
The Power Spectral Density for the 4–40 Hz frequency range of the performer participant during the baseline and the overall performance of one rehearsal and the second performance.

Following the processing of the datasets, group studies were created in order to plot and analyse in comparison:

1. the 4–40 Hz Signal Potential (μV) of each audience participant in the Time domain (ms) during the baseline, the overall performance (Figure 2), part 1 “Me” (scenes 1–3), part 2 “You/We” scene 4, and part 2 “You/We” scene 5;
2. the Power Spectral Density, which describes the distribution of power, in the 4–40 Hz frequency range of each audience participant during the baseline, the overall performance, part 1 “Me” (scenes 1–3), part 2 “You/We” scene 4, and part 2 “You/We” scene 5 (Figures 3–5);
3. the Power Spectral Density for the 4–40 Hz frequency range of the performer participant during the baseline and the overall performance (Figure 6). The recorded EEG data from the first and third performance had to be excluded, due to the repetitive disconnections that occurred in the transmission of the data from the BCI device to the computer. For similar reasons the scenes 1–3, 4, and 5 were not examined individually from the overall performance, as in the case of the audience participants.

### Audience participants' power spectral density intra-subject variability

Although the EEGLAB interactive toolbox offers automatic functions for calculating a range of statistics for the plotted data, these did not seem to reliably work in the case of the currently discussed study and for this reason manual calculations were performed. More specifically, the datapoints' values from each individual plot were extracted and the statistical analysis was performed in combination with the Microsoft Excel, MATLAB R2016b and IBM SPSS software.

The analysis, and more specifically the intra-subject variability, of the Power Spectral Density of the audience participants across all performances has shown that their Alpha power (8–13 Hz) tended in most cases to decrease during the performance compared to the baseline (pre-performance time-period) and mostly during the scene 4 of part 2 “You/We.” This is when the actress leaves the stage, two soft spots above the two audience participants are turned on and their brain-activities are interacting with the video projections. The decrease of the Alpha power has been associated by previous research (O'Connell et al., 2009) with the increase of the cognitive function of attention. Therefore, the results of the audience participants indicate that not only were they more attentive, as expected, during the performance compared to the baseline period, but particularly during part 2 “You/We.” Whereas later on (scene 5) the actress reappears on stage, addressing directly the audience, while their real-time brain-interaction gradually merges and their averaged values are controlling the colour filter applied to the live visuals (Zioga et al., 2016).

The intra-subject variability of the Power Spectral Density of the audience participants across all performances has also shown that their Lower Gamma power (25–40 Hz) tended in most cases to increase during scene 5 of part 2 “You/We” of the performance, especially compared to the overall performance. The increase of the Lower Gamma power has been associated by previous research with increased emotional engagement (Müller et al., 1999) and emotional facial information processing (Balconi and Lucchiari, 2008). Therefore, the results of the audience participants indicate that they were more emotionally engaged during scene 5 of part 2 “You/We,” when the performer was addressing directly the audience citing “moving” fragments from *On Being Ill* by Virginia Woolf (2002), while their brain-activity was jointly interacting with the video projections.

Furthermore, a one-way Analysis of Variance (ANOVA) with repeated measures and Bonferroni *post-hoc* test was performed, in order to compare the mean variability of each frequency band between the baseline period, the overall performance, scenes 1–3, scene 4, and scene 5, for each audience participant. A one-way ANOVA is the statistical method of choice, when the aim is to compare groups of three or more means at the same time and identify whether they differ significantly (Lowry, 1999). Whereas, *post-hoc* tests and pairwise comparisons are used to determine which means differ the most. The analysis confirmed that there were significant between the means differences in the Alpha Power Spectral Density [*F*_(4, 20)_ = 3.422 and *p* < 0.05], with a greater decrease occurring during scene 4 compared to the baseline period. The analysis also confirmed that there were significant between the means differences in the Lower Gamma Power Spectral Density [*F*_(4, 20)_ = 2.899 and *p* < 0.05] with a significant increase during scene 5 compared to the overall performance.

### Audience participants' inter-subject time-frequency correlation analysis

Additionally, an inter-subject 4–40 Hz time-frequency correlation analysis was performed between the audience participants of each performance. As also explained at the beginning of this section, during the processing and analysis of the data a series of challenges have been encountered. In the case of the correlation analysis the process was not possible to be completed inside the IBM SPSS software due to particular large amount—up to 725,505—datapoints. In order to overcome this problem, the datapoints' values from each individual plot were extracted and the MATLAB R2016b software was used in order to calculate the Spearman's rank correlation coefficient. The results showed that in all three performances, the correlation, ρ (rho) value, between the audience participants was greater and significant (*p* < 0.05) or highly significant (*p* < 0.001) during scene 4 of part 2 “You/We” when also their attention was increased (Table 4).

**Table 4:** Inter-subject time-frequency correlation analysis for the audience participants' 4–40 Hz.

**Event**	**Overall perform**.		**Scene 1–3**		**Scene 4**		**Scene 5**	
	**ρ (rho)**	***p*-value**	**ρ (rho)**	***p*-value**	**ρ (rho)**	***p*-value**	**ρ (rho)**	***p*-value**
1st perf.	0.002	<0.05	0.007	<0.001	−0.015	<0.001	−0.002	0.401
2nd perf.	0.008	<0.001	0.003	0.058	0.024	<0.001	0.016	<0.001
3rd perf.	0.001	0.289	−0.001	0.513	−0.009	<0.05	0.002	0.426

### Performer participant's power spectral density intra-subject variability

As in the case of the audience participants' data processing and analysis, the available automatic functions of the EEGLAB interactive toolbox for calculating a range of statistics did not seem to reliably work also with the performer participant's plotted data and for this reason manual calculations were performed. Similarly, the datapoints' values from each individual plot were extracted and the statistical analysis was performed in combination with the Microsoft Excel, MATLAB R2016b and IBM SPSS software. Also as mentioned previously, the EEG data from the first and third performance had to be excluded, due to the repetitive disconnections that occurred in the transmission of the data from the BCI device to the computer. For similar reasons the scenes 1–3, 4, and 5 were not examined individually from the overall performance, as in the case of the audience participants. However, recordings were obtained during the performer's rehearsals and these were used in the analysis.

The analysis of the intra-subject variability of the 4–40 Hz Power Spectral Density of the performer participant during one rehearsal and the second performance has shown that the Theta power (4–8 Hz) and Lower Gamma power (25–40 Hz) tended to increase significantly during the overall performance compared to the baseline period. The increase of the Theta power, and more specifically over frontal brain areas, has been associated by previous research with memory encoding (Klimesch, 1999), the active maintenance and recall of working memory representations, and the increase of “memory load in a working memory task” (Jensen and Tesche, 2002). At the same time, Gamma power has also been associated by previous research with directed attention and maintenance of working memory (Howard et al., 2003; Jensen et al., 2007).

## Discussion

### Summary and interpretation of findings

The analysis of the participants' data and the comparison of the results reveal a correlation between their answers to the questionnaires and the EEG data. More specifically, the majority of the audience participants and the performer participant across the majority of the events were able to successfully identify whether their brain-activity was interacting with the live visuals or not, and highlighted as main factors the changing colours of the visuals, part 2 “You/We” of the performance, the explanatory vignettes and the dramaturgical use of lights. At the same time, apart from the experience as a whole, the most highlighted elements that made a special impression on them include again the live visuals and the colours, part 2 “You/We” of the performance, the use of different languages and also the “moving” texts.

These results are further reinforced in comparison with the intra-subject variability of the EEG Power Spectral Density of the audience participants across all performances, confirmed also by a one-way ANOVA analysis. One the one hand, it has shown that their Alpha power (8–13 Hz) tended to decrease and therefore were more attentive during scene 4 of part 2 “You/We,” when their brain-activity was interacting with the video projections without the presence of the actress. On the other hand, it has also shown that their Lower Gamma power (25–40 Hz) tended to increase during scene 5 of part 2 “You/We,” which means that they were more emotionally engaged, while they were processing the actress' emotional facial information, when she was directly addressing them and citing “moving” texts. Additionally, the inter-subject 4–40 Hz time-frequency correlation analysis showed that the correlation between the audience participants was greater and significant or highly significant during scene 4 of part 2 “You/We.”

Furthermore, the evidenced relationship between the participants' BCI interaction awareness, the elements of special impression on them and their cognitive state during scene 4 and 5 of part 2 “You/We” can be compared to findings from studies that investigate the effect of films on the spectators' brain activity, searching for similarities in their spatiotemporal responses (Hasson et al., 2008). For example, in a study by Dmochowski et al. (2012), the results revealed peak inter-subject correlation of neural activity during arousing moments of a film, which according to the authors “reflects attention- and emotion-modulated cortical processing.” In a similar investigation in a real-life context by Jola et al. (2011), the audience participants' cortical excitability was measured while watching “a dress rehearsal of a commercial production of Sleeping Beauty, lasting 2.5 h, performed by the Scottish Ballet.” By cortical excitability the authors refer to the motor empathy of the participant's while watching the performance of the dancers. As they observed, the participants' responses “were strongly individual” and their “cortical excitability decreased with time,” which could be due to different reasons, such as the long duration of the play, but nevertheless highlights the importance of the use of time in a dance performance. Relevant observations, such as “fatigue and distraction” (Jin et al., 2011), are also commonly observed in BCI studies with long duration, while researchers are currently investigating new experimental paradigms that will help address these issues. Following this thread, we argue that the results of the currently discussed study, more in particular the increase of the factors associated with the audience participants' attention, emotional engagement and facial processing during the last two scenes of the performance, serve as a strong evidence of the importance of the directing strategy, dramaturgy, and narrative structure. These methods and strategies can make effective use of the performance time and lead the audience's perception and cognitive state during the different stages of the work.

Last but not least, the analysis of the intra-subject variability of the 4–40 Hz Power Spectral Density of the performer participant has shown that the Theta power (4–8 Hz) and Lower Gamma power (25–40 Hz) tended to increase significantly during the overall performance compared to the baseline period, which is consistent with the recall of working memory representations and the increase of cognitive load.

### Limitations

One of the important limitations in “*Enheduanna—A Manifesto of Falling” Live Brain-Computer Cinema Performance*, as in the case of other similar studies (Jola et al., 2011; Eaton et al., 2015), has been the data collection in terms of quantity. Due to the demanding logistics of the events (allocation of space, costs etc.), it has not been made possible to repeat the performance several times nor include a large number of audience participants in each event. With six audience participants and one performer participant one can argue that the sample of the data is non-representative. While at the same time, the comparison between participants of different events is difficult, since the duration of live events, no matter how well coordinated they are, can be even slightly different due to possible delays or differences in the transition from scene to scene. However, the results not only provide strong indications and insight on the perception, cognition, and engagement of spectators and performers during a live mixed-media performance, but they can also inform future development and encourage similar studies in real-life settings.

### The live brain-computer cinema performance as a neuroscientific experiment in a real-life context and future work

*Enheduanna—A Manifesto of Falling* (Zioga and Katsinavaki, 2015), the currently discussed live brain-computer cinema performance, is a new format of interactive work that combines live cinema and the use of BCIs, but also a neuroscientific experiment in a real-life context. As mentioned in section Introduction, in recent years in the fields of neuroscience and experimental psychology has emerged a new and increasing interest in studying the mechanisms, dynamics and processes of the interaction between multiple subjects and their brain-activity and even more in a real-life setting, away from the lab. Although until recently the majority of the scientific research was realised by presenting to the participants small fragments of audio-visual material and employing event-related designs, it is now acknowledged that in natural settings the brain-activity is rather continuous and transient (Dmochowski et al., 2012). While, recent studies are already using new calculation methods, such as the “practical bit rate” vs. the “raw bit rate,” in order to “estimate the speed” of BCI systems in “a reasonable world setting” (Jin et al., 2011). For this reason, the need of studies in real-life contexts has also been identified (Jin et al., 2014), including free viewing of films and live performances. As in the study by Jola et al. (2011), the currently discussed live brain-computer cinema performance makes a claim about live experiences and experiments outside the laboratory and contributes on new hypotheses about the effects of the length of time, but also the role of the directing strategy, dramaturgy and narrative structure on the audience's perception, cognitive state, and engagement. At the same time, it informs other relevant discussions of the use of BCIs as query tools in the frame of Practice as Research (PaR) (Aparicio and Cadiz, 2017).

The results of the EEG data analysis can also lead to further investigations. More specifically, new studies with increased number of audience participants could provide the initial results with statistical significance. Whereas, additional studies could look into the effect of real-life conditions, like in scene 5, when the performer by directly addressing the audience participants probed the increase of their emotional engagement and emotional facial information processing. For the above reasons, we believe that this study is a step toward gaining greater understanding of neuromodulation in performers and audiences alike and its potential applications; but it also serves as evidence that interdisciplinary research not only can contribute to the advancement of the different fields involved, but can also result in new observations, not possible to be made in isolation.

## Author contributions

PZ: conceptualised and conducted the study, acquired the data and wrote the manuscript; PZ, FP, PC, and MM: contributed to the methodological design of the study; PZ, KS, and FP: carried out the data analysis and the interpretation of the results. All authors contributed to the critical revision and editing of the manuscript.

### Conflict of interest statement

The authors declare that the research was conducted in the absence of any commercial or financial relationships that could be construed as a potential conflict of interest.
